# Experimental Tools to Study Molecular Recognition within the Nanoparticle Corona

**DOI:** 10.3390/s140916196

**Published:** 2014-09-02

**Authors:** Markita P. Landry, Sebastian Kruss, Justin T. Nelson, Gili Bisker, Nicole M. Iverson, Nigel F. Reuel, Michael S. Strano

**Affiliations:** Department of Chemical Engineering, Massachusetts Institute of Technology, 77 Massachusetts Ave. Cambridge, MA 02139, USA; E-Mails: markita@mit.edu (M.P.L.); kruss@mit.edu (S.K.); jtnelson@mit.edu (J.T.N.); bisker@mit.edu (G.B.); niverson@mit.edu (N.M.I.); reuel@mit.edu (N.F.R.)

**Keywords:** optical sensors, molecular recognition, fluorescence, single-molecule microscopy, neurotransmitters, corona phase, carbon nanotubes

## Abstract

Advancements in optical nanosensor development have enabled the design of sensors using syntheticmolecular recognition elements through a recently developed method called Corona Phase MolecularRecognition (CoPhMoRe). The synthetic sensors resulting from these design principles are highly selective for specific analytes, and demonstrate remarkable stability for use under a variety of conditions. An essential element of nanosensor development hinges on the ability to understand the interface between nanoparticles and the associated corona phase surrounding the nanosensor, an environment outside of the range of traditional characterization tools, such as NMR. This review discusses the need for new strategies and instrumentation to study the nanoparticle corona, operating in both *in vitro* and *in vivo* environments. Approaches to instrumentation must have the capacity to concurrently monitor nanosensor operation and the molecular changes in the corona phase. A detailed overview of new tools for the understanding of CoPhMoRe mechanisms is provided for future applications.

## 1. Introduction

In recent years, the use of nanomaterials for sensing applications has undergone rapid growth, in particular the development of high-sensitivity and high-specificity sensors for processes occurring at the molecular scale [1]. While many such sensors are electrochemical in nature [2], there are distinct advantages to the development of optical sensors for molecular targets, particularly when information is required in the spatial domain. Recent advances in the development of optical sensors of molecular targets have relied on fluorescent nanomaterials for the transduction of the sensor response. Nanomaterials have unique optical, chemical, and mechanical properties that make them useful for such applications [3,4]. In particular, fluorescent semiconducting single-wall carbon nanotubes (SWCNT) are excellent materials to use as the signal transduction element of optical sensors, due to their long fluorescent lifetimes, sensitivity, and photostability [5]. Additionally, for use in biological sensing applications, SWCNT emit in an optical window (near infrared) where tissues, cells, blood, and other biological samples are transparent [6].

Specific functionalization of SWCNT with polymers (biopolymers or synthetic polymers), that necessarily form a corona surrounding the nanoparticle, can impart molecular recognition capabilities to the SWCNT transducer, such that the SWCNT-polymer conjugate produces a signature SWCNT-mediated intensity shift in the presence of a specific analyte molecule [7–9]. The engineering of novel molecular recognition entities on carbon nanotube surfaces has recently enabled the optical detection of analytes using an approach called Corona Phase Molecular Recognition (CoPhMoRe). This powerful technique benefits from the rational design of SWCNT-based sensors that produce high-specificity optical signals. However, until recently, there has been a dearth of tools to study the fundamental corona phase-nanotube interactions that lead to these high-specificity and high-selectivity signals. Conventional optical tools have insufficient overlap to study both polymers and nanomaterials simultaneously [10,11], which is becoming increasingly necessary to advance fields of research that hinge on polymer-nanoparticle interactions such as the design of SWCNT-based sensors [12,13]. This hampers the ability to predict relevant parameters leading to proper design of sensors and leaves researchers ill-equipped to design novel sensors and other nanoparticle-based sensing and diagnostic tools [14].

Tools to study sensors on a molecular scale have emerged to fill the need for concurrent characterization of SWCNT and their corona phases that provide the molecular recognition for sensing applications. For instance, for the concurrent optical study of SWCNT and biopolymers, a novel microscope has been designed by merging two commonly used microscopy techniques. Together with an emergent development in laser technology, this novel instrument has the capacity to study both nanoparticles and individual polymers simultaneously with spatial and temporal resolution that is appropriate for nanoscale interactions of the corona phase around SWCNT that impart sensing capabilities to nanosensors. Microscopy tools such as this are crucial for the development of sensors for use *in vitro* and *in vivo*.

## 2. Experimental Section

### Hybrid Microscope for Single-Molecule Visualization of SWCNT-Based Sensors

A powerful tool to study individual molecules and their interactions *in vitro* [15] and *in vivo* [16] is single-molecule Total Internal Reflection Fluorescence Microscopy (sm-TIRF). Imaging with sm-TIRF combines nanometer spatial resolution and millisecond temporal resolution with the ability to simultaneously detect hundreds of multi-component molecular complexes interacting in a microfluidic chamber using visible fluorescence microscopy [17]. Conversely, nanomaterials such as SWCNT are commonly studied using near-infrared (nIR) epifluorescence microscopy, due to their intrinsic nIR fluorescence (nIRF) [18]. While biophysical techniques such as sm-TIRF only report on the state of the biomolecules under study, monitoring the intrinsic fluorescence of SWCNT in the nIR only reports on the electronic changes incurred by the SWCNT itself. Recent microscopy advancements have successfully combined both sm-TIRF and nIR microscopy into a single instrument, a nIRF TIRF microscope, with the capacity to study both SWCNT and the SWCNT corona phase simultaneously. This microscope provides the spatial and temporal resolution required for characterizing nanoscale interactions that occur between the SWCNT and the corona polymer for a better understanding of SWCNT-based sensing.

Several practical considerations must be undertaken to successfully image both the SWCNT and the corona phase with a nIRF TIRF microscope. First, most SWCNT-based sensors are comprised of a mixture of different SWCNT chiralities that have a broad distribution of excitation and emission maxima. The visible fluorescence tags commonly used to visualize the SWCNT corona vary just as much in excitation and emission characteristics. Therefore, the nIRF TIRF must be able to excite a SWCNT-based sensor sample across a broad range of wavelengths. Second, the nIRF TIRF must capture the broad range of emission wavelengths produced by fluorophores (visible emission) and SWCNT (nIR emission) simultaneously. Third, to capture the processes of molecular recognition between the SWCNT sensor and its analyte, optical imaging of the SWCNT must be performed with high spatial (nanometer) and temporal (millisecond) resolution while monitoring both the SWCNT signal and the corona phase signal. Proper incorporation of these design rules enables the study of the corona phase tagged with visible fluorophores concurrently with changes in SWCNT nIR fluorescence.

To excite over a wide range of SWCNT chiralities and visible fluorophores, the nIRF TIRF microscope uses a newly available light source that combines the power and coherence of a standard monochromatic laser with the excitation wavelength range of a white lamp. Recent advances in laser diode technology have enabled high-power and high-coherence supercontinuum white light generation by coupling a picosecond-pulsed nIR laser into a nonlinear photonic crystal to generate a coherent supercontinuum wavelength output [19]. While traditional sm-TIRF microscopes are built using one or several monochromatic lasers that specifically excite fluorophores with discrete excitation peaks [20], the nIRF TIRF microscope uses a supercontinuum laser coupled to a tunable wavelength and bandwidth filter to excite samples with excitation light from 480 nm to 800 nm. The resulting excitation wavelength range of enables visualization of dozens of visible fluorophores, and all excitation peaks within a typical HiPco SWCNT sample [21]. This emergent laser technology has recently been employed for TIRF microscopy or for the study of nano-bio interactions [22,23].

**Figure 1.**
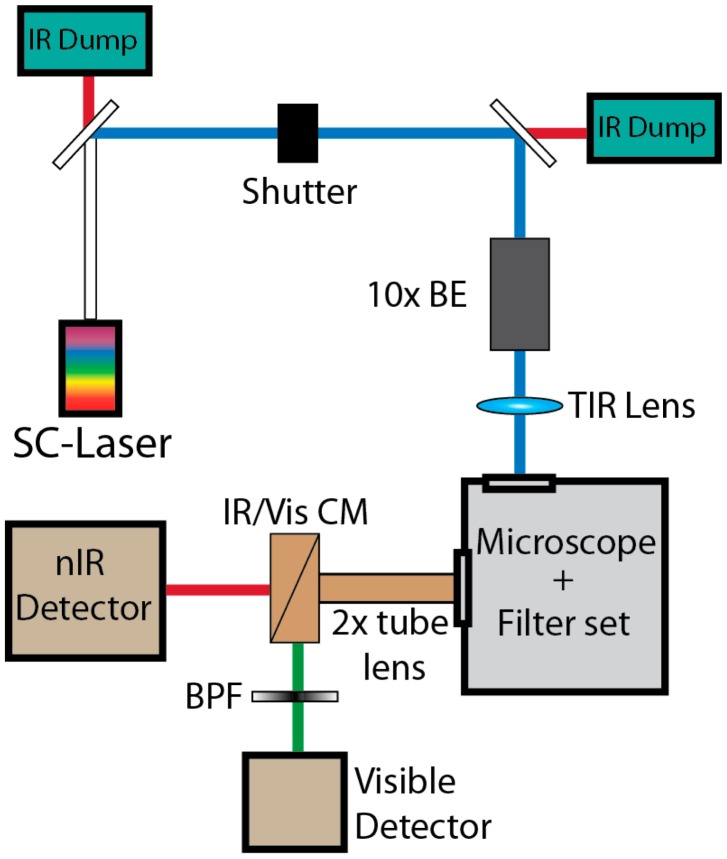
Instrument design. A Supercontinuum (SC) laser is attenuated with a neutral density (ND) filter. The beam undergoes 10× beam expansion (BE) to overfill the microscope objective for beam-steering via a plano-convex lens. A collimating lens in the microscope body collects emission while a 2× tube lens expands the image to fill the imaging plane of two detectors. A cold mirror (CM) separates nIR and visible light. Visible path: Signal is filtered with a band-pass filter (BPF) onto an EM-CCD camera in a 512 × 512 pixel imaging area. *nIR path*: nIR emission is directed onto a nIR detector at the same imaging plane as the visible detector (with permission from [24]).

To visualize across a broad range of both visible (fluorophore-tagged corona phases) and nIR emission (SWCNT emission) wavelengths, the nIRF TIRF microscope emission path is designed for concurrent sampling of visible and nIR sample emissions. At the emission port, a cold mirror is used to separate nIR from visible emission wavelengths, and directs nIR emission into a nIR camera. Visible emissions follow an emission path similar to that of a traditional sm-TIRF setup, allowing concurrent monitoring of two distinct visible emission wavelengths (Figure 1). The combined capability to excite (450 nm–800 nm) and monitor (900 nm–1800 nm) across a broad range of visible and nIR wavelengths has made the nIRF TIRF microscope and excellent tool to extend the understanding of how SWCNT coronae provide selectivity and sensitivity to SWCNT-based optical sensors for several applications, as detailed below.

## 3. Results and Discussion

### 3.1. Conformational Dynamics in the Corona Phase of Nanobiosensors

The importance of molecular recognition spans across many fields, particularly for mechanisms involving biological structures such as antibodies. Naturally occurring antibody-antigen pairs have been used for numerous applications [25,26], principally for diagnostics and sensing. However, identifying and isolating an antibody for a particular biological molecule is often costly and time-consuming. Recent work has succeeded in developing a synthesis platform for the design synthetic sensors, based on the principle of SWCNT CoPhMoRe. The design of sensors with CoPhMoRe is based on the adsorptive properties of engineered heteropolymers to SWCNT, which create a unique SWCNT corona with molecular recognition properties for a target analyte [24].

**Figure 2.**
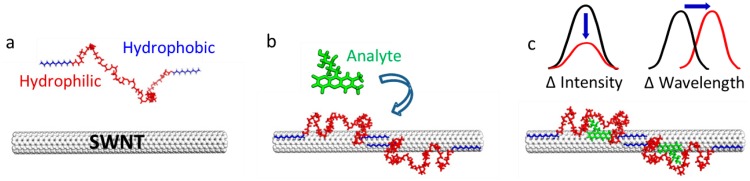
Principle of CoPhMoRe. (**a**) A polymer with hydrophobic and hydrophilic parts adopts a specific conformation when (**b**) adsorbed to the nanotube, thereby enabling analyte-specific binding. (**c**) Selective analyte binding leads to a wavelength or intensity change in SWCNT fluorescence (with permission from [24]).

Molecular recognition hinges upon proper orientation of interacting molecules in three-dimensional space, and the subsequent creation of a unique binding interface between the molecules. In this recent work, process of biomolecular recognition was mimicked on a SWCNT surface, to create synthetic sensors. This was accomplished by designing a library of heterogeneous synthetic heteropolymers that created unique binding sites when adsorbed to the surface of a SWCNT. These heteropolymers were designed and synthesized to contain alternating hydrophilic-hydrophobic domains that adsorbed noncovalently onto a SWCNT surface such that the hydrophobic segments adsorb to the SWCNT and the hydrophilic segments extend into the aqueous environment and form a molecular recognition “corona” to bind a particular analyte (Figure 2a).

These heteropolymers were then screened against an analyte library, resulting in certain sensor-analyte pair ‘hits’ that can serve as sensors. These sensors provide a unique high-selectivity and high-sensitivity near-infrared signal upon binding of their analyte, as demonstrated by the selective detection of three SWCNT sensor-analyte pairs identified as examples of CoPhMoRe: estradiol hormone was detected by a rhodamine isothiocyanate-difunctionalized poly(ethylene glycol)-SWCNT complex (RITC-PEG-RITC), thyroxine hormone was detected by an Fmoc L-phenylalanine polymer-SWCNT complex (Fmoc-Phe-PPEG8-SWCNT), and essential vitamin riboflavin was detected by boronic acid substituted phenoxy-dextran-wrapped SWCNT (BA-PhO-Dex-SWCNT). Chemical mutations of the hydrophobic anchor segments for each polymer resulted in a loss of sensor sensitivity, selectivity, or both. In contrast, lengthening the hydrophilic PEG segment of the RITC-PEG-RITC polymer had little effect on the sensor response profile for hormone estradiol. Therefore, synthetic sensors produced with CoPhMoRe likely rely primarily on the interaction between the hydrophobic anchor segment of the polymer, and the nanotube surface.

In this work, the authors used a nIRF TIRF microscope to probe the single-molecule dynamics of the SWCNT sensor coronae for sensors developed with the CoPhMoRe technique. Quenching of organic fluorophores occurs as a function of proximity to the surface of SWCNT [27], thereby creating a fluorescent ruler to quantify the degree of polymer desorption. Fortuitously, the RITC domain of the RITC-PEG-RITC-SWCNT sensor for estradiol is a fluorophore (with ex/em = 543 nm/580 nm) providing an optical signal with which to probe the RITC-PEG-RITC polymer structure with respect to the carbon nanotube. Dim RITC fluorophore signals are expected for polymer hydrophobic “anchors” that remain adsorbed to the SWCNT surface, whereas brightening of the RITC fluorophore is expected if these “anchor” domains desorb if perturbed from the SWCNT surface.

The RITC-PEG-RITC SWCNT sensors were first surface-immobilized within a microfluidic device and mounted onto the nIRF TIRF microscope. Both the nIR fluorescence of the SWCNT and the fluorescence of the RITC fluorophores were monitored through each of the nIRF TIRF channels. Upon addition of 500 μM estradiol in the sample chamber, the nIR fluorescence intensity of the SWCNT decreased as expected for the sensor response. However, the number of fluorescent RITC fluorophores after exposure to 500 μM estradiol increased from 271.7 ± 4.5 to 304.5 ± 4.4 (mean ± SE). The sensor quenching and RITC fluorescence increase both occurred simultaneously. The concurrence of nIR and visible signals strongly suggest that the nIR RITC-PEG-RITC-SWCNT sensor response to estradiol is mediated by a desorption of the RITC anchor from the SWCNT surface.

### 3.2. A New Tool for Nanoscale Optical Detection of Neurotransmitters

Nanoscale optical sensors are particularly beneficial for fields of study where high-resolution spatial and temporal information is required to fully characterize the system. Neuroscience, and the study of neurotransmitter release, is a good example of a system for which nanoscale optical sensors can be particularly beneficial. The ability to monitor and diagnose prevalent neurodegenerative diseases is limited by the scale at which the fundamental processes that cause the transition into a disease state can be observed. In the case of neurodegenerative diseases, the pathways that fail are often the transfer of neurochemical signals from neuron to neuron. Therefore, monitoring the concentration profile of neurotransmitters is of intrinsic interest for neuroscience and necessary to fully understand neuronal networks and how the brain works to better understand neurotransmitter malfunction [28]. Several other neurological diseases such as schizophrenia are also tightly linked to particular neurotransmitters. However, the etiology of these diseases remains largely unknown, particularly in their relation to neurotransmitter release, and the chemical intricacies that lead to poor neurotransmission [29]. Many neurotransmitters are small molecules and share structural homologies with each other and other interfering substances in the brain or in cell culture. Furthermore, the number of molecules that are released in exocytotic events is relatively small, on the order of 100–10,000 molecules, and often occur within milliseconds [30]. Finally, the most prominent release sites, *i.e.* synaptic clefts, are very small (300 × 300 × 20 nm) and not easily accessible by macroscopic probes [31]. These intrinsic qualities of neuronal networks indicate that neurotransmitter sensors and probes should be sensitive, selective, reversible, small (on the nanoscale), respond to a neurotransmitter within milliseconds, and transduce signals optically in a wavelength window that will not interfere with neuronal cell samples or brain tissue.

**Figure 3.**
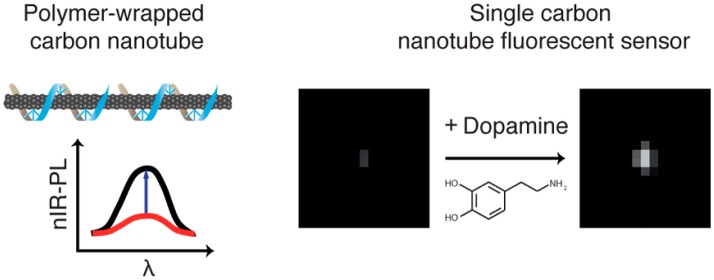
A polymer (blue) wraps a SWCNT, and provides a unique molecular recognition binding site for dopamine. Upon exposure to dopamine, the SWCNT near-infrared fluorescence signal increases up to 400% for a single SWCNT. The box dimension is 5 × 5 μm [32].

As an additional benefit of single-molecule imaging, authors were able to colocalize the SWCNT nIR and RITC visible signals. Several instances of the appearance of RITC fluorescence along the length of individual SWCNT were observed, and the colocalization probability increased significantly after the addition of the estradiol analyte. The colocalization between individual RITC fluorophores and SWCNT is a direct observation of molecular-scale sensing with CoPhMoRe, and can be extended for the study of other nanoscale sensors that rely on corona-phases for molecular recognition. Recent work has yielded CoPhMoRe nanosensors for the detection of neurotransmitters at length and time scales appropriate of *in vivo* measurements [32]. In particular, a (GT)15 DNA corona provides a selective response to neurotransmitter dopamine, when wrapped around SWCNT. Upon addition of 100 μM dopamine, the near-infrared fluorescence of these nanosensors can triple, within milliseconds of dopamine exposure [32] (Figure 3). These nanosensors are reversible, and can also detect dopamine in the presence of common interfering chemical and structural analogs, such as L-DOPA, uric acid, homovanillic acid, L-tryptophan, L-tyrosine and L-phenylalanine. This optical turn-on sensor for dopamine shows promise as a new tool for neuroscience and could provide completely new insights into synaptic transmission.

**Figure 4.**
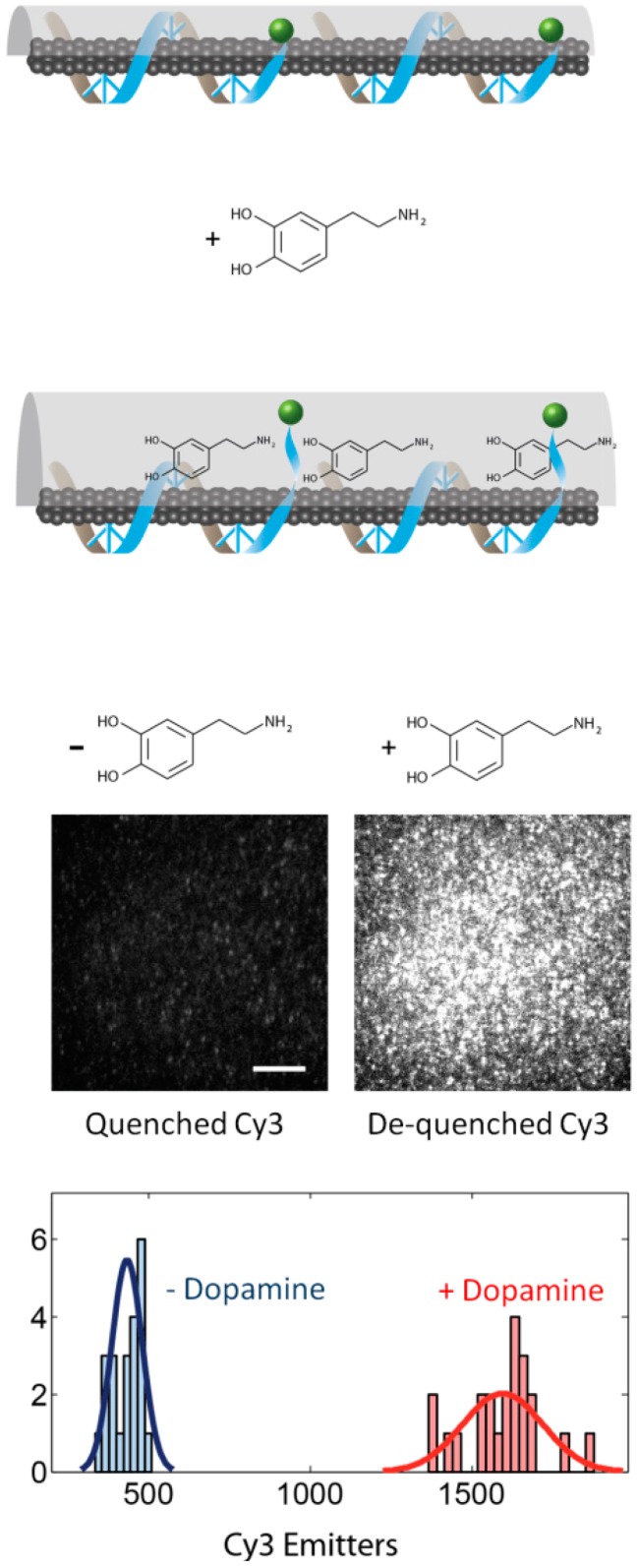
A schematic of the model suggests the displacement of the (GT)15 polymer enables dopamine to access the SWCNT corona to modulate the nIR intensity. Surface-immobilized (GT)15-Cy3-SWCNT show an increase in Cy3 fluorescence after dopamine addition, suggesting desorption of the Cy3 from the SWCNT surface [32].

In this work, the molecular-scale characterization of the (GT)15 DNA-SWCNT sensor for dopamine was also probed with the nIRF TIRF. In this case, the (GT)15 polymer is not inherently fluorescent, but a Cy3 fluorophore was synthesized onto the 3′ end of the (GT)15 DNA to allow a query of the polymer terminus conformation at the single-molecule level. In an assay similar to the one used to probe the RITC-PEG-RITC SWCNT sensor for estradiol, the (GT)15-Cy3 DNA-SWCNT sensor was immobilized within a microfluidic channel and imaged at the single-molecule level with a nIRF TIRF microscope. As expected, upon perfusion of 100 μM dopamine, the nIR signal of the (GT)15-Cy3 DNA-SWCNT sensor brightened. The visible channel of the nIRF TIRF reveals the conformation of the (GT)15 polymer on the SWCNT: upon perfusion of 100 μM dopamine, the terminal Cy3 brightens, suggesting a desorption of the fluorophore from the SWCNT surface. The Cy3 brightening occurs instantaneously upon exposure to dopamine, as does the SWCNT nIR response [32] (Figure 4). Consequently, the authors conclude that the nIR dopamine nanosensor response is accompanied by the displacement of the (GT)15 polymer from the SWCNT surface. While these results are useful for understanding the mechanism by which optical nanosensors detect target molecules, this approach can also be used to elucidate design principles for the synthesis of more robust nanosensors. Future nanosensors can be designed with better precision and molecular recognition for their targets based on the ability to probe association of the polymer with the SWCNT at the single-molecule scale.

### 3.3. Portable Microscopic Tools for Sensor Imaging

While the nIRF TIRF microscope enables molecular characterization of nanoscale optical sensors, there is a growing need for inexpensive and compact instrumentation for biological detection. This is especially crucial for applications such as real-time bioprocess monitoring and high-throughput screening where molecular detail may be unnecessary. Few practical options exist for research tools which can monitor biomolecular interactions without size and capital intensive equipment. Recent work has demonstrated such inexpensive and compact instrumentation for detection of human IgG and dopamine using fluorescent SWCNT-based biosensors. The detection platform is based on an inverted microscope but uses small and inexpensive optical components. A high-power 565 nm LED replaces the need for a laser excitation source. Similarly, an electronically amplified InGaAs photodetector is used in place of a liquid nitrogen-cooled InGaAs detector which is often needed to image nIR fluorophores. With this tool, label-free, real-time detection of human IgG is demonstrated, as well as determination of kinetic and equilibrium binding constants. This platform can readily be extended to the detection of other proteins and molecules, including targets of CoPhMoRe-based sensors.

**Figure 5.**
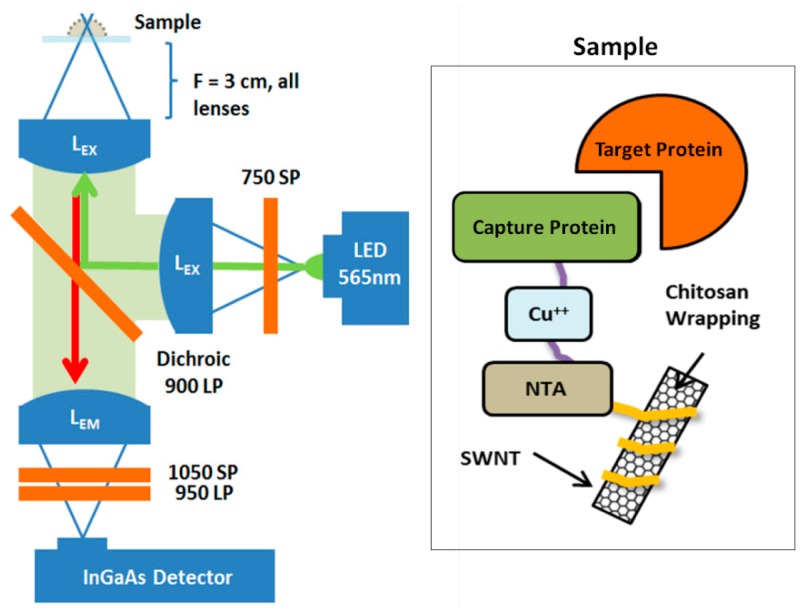
Inverted microscope designed to capture changes in SWCNT sample near-infrared intensity from the sample plane. This protable design allows label-free real-time detection of bioanalytes. Binding of the target protein (**orange**) to the capture protein (**green**) modulates the SWCNT near-infrared emission, captured by the microscope.

A black box *in vivo* imaging system is another common and compact option for collection of fluorescence data, but for the detection of the SWCNT-based biosensors, custom modifications to commercial *in vivo* fluorescence imaging systems are necessary. Recent work showed the viability of an *in vivo* biosensor when a high power laser, 1 W at 561 nm, was used to excite the samples and a liquid crystal filter was utilized to collect emission spectrum at varying wavelengths (Figure 5). This unique collection technique and the nIRF of the SWCNT enable real-time visualization of a SWCNT-based sensor for nitric oxide, free from autofluorescence or background noise which is common for *in vivo* optical sensing. To perform these measurements, an emission spectrum is gathered for each pixel in the frame, and an algorithm analyzes each spectrum to determine the percent of SWCNT fluorescence, background tissue, and autofluorescence noise at each of these points, delivering quantifiable values of the SWCNT fluorescence signal that can then be monitored in real time as the biosensor reacts to analytes. A recent application of this imaging system was used to detect the perfusion of nanoparticles in living plant tissues, clearly separating the near-infrared emission of nanoparticles from the autofluorescence of chloroplasts [33]. Another application of this imaging approach enables real-time detection of reactive oxygen species within living mice. With the animal receiving isoflurane gas from an anesthesia machine, the synthetic sensor was continually monitored for many hours. Due to the quantifiable and reproducible nature of the image collection system, the animal could be monitored at discrete time points and returned to normal housing between testing, over the course of over a year [34]. Future applications would benefit from theoretical design tools that provide the necessary grounds for the optimization of an implantable *in vivo* affinity sensor, and would allow for continuous, real time, monitoring of transient analyte concentrations in blood or in tissue [35].

### 3.4. Characterization Tools for Nanoparticle Corona Phases

To date, most nanobiosensors are designed mainly by a common, generic approach: A known biological recognition element such as an antibody is immobilized onto a nanoparticle, which may or may not be functionalized with additional organic groups. If the analyte binds the biological recognition element, physicochemical properties such as the dielectric constant around the nanoparticle can be changed, and affect the optoelectronic properties of the nanomaterial [36], for example. However, even if the analyte binds to this recognition motif, signal transduction can be poor, which impairs or hampers the use of the resulting sensor. Rational chemical design of the corona phase of nanobiosensors is desired, but there is a dearth in understanding the mechanisms that underlie analyte recognition and signal transduction in the corona phase. Therefore, characterization tools such as the above mentioned nIRF-TIRF microscope are essential to advance rational design and performance of biosensors for various applications. Table 1 gives an overview on existing methods that can shed light into the structure, composition and conformational dynamics of the organic corona phase around nanoparticles. We focus on methods that can be used with carbon nanotube-based fluorescent sensors. This summary of methods for elucidation of the organic corona phases around carbon nanotubes is also applicable to other nanoparticle-based systems.

The methods listed in Table 1 can provide valuable but different and often complementary information about the organic phase around nanoparticles. An important consideration when selecting an approach to study nanoparticle conjugates is whether single-nanoparticle resolution is desired, or if measurements of bulk properties are sufficient. For example, AFM can be used to visualize the height profile of a single carbon nanotube and its organic phase, often in solid phase. In contrast, absorption measurements are typically done in bulk but provide important information about the dielectric environment around the nanoparticle in solution. Another consideration is chemical or physical contrast. AFM provides primarily mechanical and topological information while mass spectrometry and NMR provide insights into the composition and chemical structure of the organic phase. Other methods such as fluorescence spectroscopy and microscopy do provide indirect measures about the corona phase. For instance, analyte binding could affect the conformation of the corona phase, which could further change fluorescence quantum yields of the nanomaterial or could shifts features in the spectrum. To use fluorescence measurements, a calibration is often needed to correlate structural dynamics to variations in fluorescence quantum yield in the fluorescence spectrum.

One of the most promising techniques for visualizing nanostructured materials without air-drying a sample is cryogenic transmission electron microscopy (cryo-TEM). This approach is based on an instantaneous freezing of a liquid sample, followed by transmission electron microscopy imaging [46] of the matrix. The main advantage of this method is that it reveals information at the nanoscale about liquid samples in their native hydrated phase, without artifacts caused by sample drying [47]. Combined with powerful 3-dimenssional reconstruction computational algorithms, the structures of monodispersed biological molecules can be solved with sub-nanometer resolution [48]. In the case of SWCNT suspensions, cryo-TEM imaging can be utilized to determine whether the nanotubes are individually suspended rather than bundled in solution [42]. The ability of amphiphiles to successfully suspend and debundle SWCNT can be also assessed by small-angle neutron scattering (SANS), where the form of the scattering intensity as a function of the scattering vector can differentiate between individual rod-like tubes, and more complex structures [49].

Optical circular dichroism (CD) measurement detects the difference in absorption between left and right circular polarized light [50]. Although SWCNT have left or right handedness [51], giving them circular dichroism activity, it is difficult to detect their CD spectrum since they are synthesized in racemic mixtures [40]. When SWCNT are suspended in solution, CD measurements can thus reveal whether the polymer that forms the corona phase gives rise to an uneven distribution of left handed and right handed polymer-SWCNT complexes, as in the case of DNA wrapped SWCNT [40]. Moreover, CD measurements can confirm that if nanotubes are suspended using an optically active polymer, such as polyaniline, the polymer-nanotube complex retain that optical activity [52]. Measuring the intrinsic CD spectra of SWCNT is possible only on the single molecule level, with a single nanotube and a custom experimental system [53].

In summary, determining the structure and dynamics of the molecular phase around nanoparticles (*i.e.*, the corona) with high spatial and temporal resolution in an aqueous environment is extremely challenging. Each method can shed light on a different aspect of these complex constructs, and one must combine several approaches in order to gain true insight about the organic corona phase, and allow for better mechanistic understanding and design rules for nanobiosensors.

## 4. Conclusions/Outlook

Emerging microscopy platforms will aid in the design of future synthetic optical sensing tools based on SWCNT-polymer hybrids, which is promising for areas in need of high-specificity sensors, such as label-free detection of analytes, nanoparticle-based diagnostic tools, and nanoscale therapeutics. Recent work has shown that SWCNT can be used as the basis for the development of synthetic sensors based on the 3-dimensional spatial confinement of a heteropolymer on the SWCNT surface [24]. These SWCNT-heteropolymer hybrids, called synthetic nanotube-templated sensors are in essence synthetically engineered antibody-like constructs with high-specificity for a particular analyte by providing a strong near-infrared optical signal in the presence of the target molecule. The biopolymer serves a dual purpose: To bestow high-selectivity towards a particular analyte molecule, and to mask the hydrophobic and potentially toxic character of the SWCNT. The interactions between these polymers and the SWCNT are the key to understanding how to design more selective sensors. New microscopic tools enable concurrent monitoring of SWCNT fluorescence and the behavior of the heteropolymer, which can be used to design and characterize synthetic sensors for several applications. Potential uses of these nanotube-based sensors include low-cost medical diagnostics, biodefense screening tools, and rapid methods to test structure-function relationships of biological molecules.

## Figures and Tables

**Table 1.** Summary of methods commonly used to elucidate the structure of the corona phase around carbon nanotube sensors.

Method	Example
Mass spectrometry	Composition of the protein corona around SWCNTs [37]
AFM	Periodic structure of DNA wrapped around SWCNTs [38]
NMR	Monitoring functionalization and composition of SWCNTs [39]
Circular Dichroism	DNA structure and chirality around SWCNTs [40]
Absorption spectroscopy	Dielectric environment around SWCNTs [41,42]
Fluorescence spectroscopy/microscopy	Quantification of SWCNT fluorescence changes and correlation with structure of the organic phase [24,32]
Functional groups assays	Identification of functional groups in the organic phase around SWCNT [43]
Cryogenic transmission electron microscopy (cryo-TEM)	Characterization of thermosensitive corona shell around spherical polystyrene particles [44]
Small-Angle Neutron Scattering (SANS)	Quantification of amphiphilic block copolymers adsorption onto SWCNT surface [45]

## References

[1] Wang Q.H., Bellisario D.O., Drahushuk L.W., Jain R.M., Kruss S., Landry M.P., Mahajan S.G., Shimizu S.F.E., Ulissi Z.W., Strano M.S. (2014). Low Dimensional Carbon Materials for Applications in Mass and Energy Transport. Chem. Mater..

[2] Jacobs C.B., Peairs M.J., Venton B.J. (2010). Review: Carbon nanotube based electrochemical sensors for biomolecules. Anal. Chim. Acta.

[3] Langer R., Tirrell D.A. (2004). Designing materials for biology and medicine. Nature.

[4] Rosi N.L., Giljohann D.A., Thaxton C.S., Lytton-Jean A.K.R., Han M.S., Mirkin C.A. (2006). Oligonucleotide-modified gold nanoparticles for intracellular gene regulation. Science.

[5] Barone P.W., Strano M.S. (2006). Reversible control of carbon nanotube aggregation for a glucose affinity sensor. Angew. Chem. Int. Ed..

[6] Boghossian A.A., Zhang J., Barone P.W., Reuel N.F., Kim J.H., Heller D.A., Ahn J.H., Hilmer A.J., Rwei A., Arkalgud J.R. (2011). Near-infrared fluorescent sensors based on single-walled carbon nanotubes for life sciences applications. ChemSusChem..

[7] Reuel N.F., Ahn J.H., Kim J.H., Zhang J., Boghossian A.A., Mahal L.K., Strano M.S. (2011). Transduction of glycan-lectin binding using near-infrared fluorescent single-walled carbon nanotubes for glycan profiling. J. Am. Chem. Soc..

[8] Zhang J.Q., Boghossian A.A., Barone P.W., Rwei A., Kim J.H., Lin D.H., Heller D.A., Hilmer A.J., Nair N., Reuel N.F. (2011). Single Molecule Detection of Nitric Oxide Enabled by d(AT)(15) DNA Adsorbed to Near Infrared Fluorescent Single-Walled Carbon Nanotubes. J. Am. Chem. Soc..

[9] Pender M.J., Sowards L.A., Hartgerink J.D., Stone M.O., Naik R.R. (2006). Peptide-mediated formation of single-wall carbon nanotube composites. Nano Lett..

[10] Campbell J.F., Tessmer I., Thorp H.H., Erie D.A. (2008). Atomic force microscopy studies of DNA-wrapped carbon nanotube structure and binding to quantum dots. J. Am. Chem. Soc..

[11] Sapsford K.E., Tyner K.M., Dair B.J., Deschamps J.R., Medintz I.L. (2011). Analyzing nanomaterial bioconjugates: A review of current and emerging purification and characterization techniques. Anal. Chem..

[12] Nel A.E., Madler L., Velegol D., Xia T., Hoek E.M., Somasundaran P., Klaessig F., Castranova V., Thompson M. (2009). Understanding biophysicochemical interactions at the nano-bio interface. Nat. Mater..

[13] Shvedova A.A., Kagan V.E., Fadeel B. (2010). Close encounters of the small kind: Adverse effects of man-made materials interfacing with the nano-cosmos of biological systems. Annu. Rev. Pharmacol. Toxicol..

[14] Hauck T.S., Ghazani A.A., Chan W.C. (2008). Assessing the effect of surface chemistry on gold nanorod uptake, toxicity, and gene expression in mammalian cells. Small.

[15] Reck-Peterson S.L., Yildiz A., Carter A.P., Gennerich A., Zhang N., Vale R.D. (2006). Single-molecule analysis of dynein processivity and stepping behavior. Cell.

[16] Kural C., Kim H., Syed S., Goshima G., Gelfand V.I., Selvin P.R. (2005). Kinesin and dynein move a peroxisome *in vivo*: A tug-of-war or coordinated movement?. Science.

[17] Jain A., Liu R., Ramani B., Arauz E., Ishitsuka Y., Ragunathan K., Park J., Chen J., Yang K.X., Ha T. (2011). Probing cellular protein complexes using single-molecule pull-down. Nature.

[18] Tsyboulski D.A., Bachilo S.M., Weisman R.B. (2005). Versatile visualization of individual single-walled carbon nanotubes with near-infrared fluorescence microscopy. Nano Lett..

[19] Telford W.G., Subach F.V., Verkhusha V.V. (2009). Supercontinuum white light lasers for flow cytometry. Cytometry A.

[20] Roy R., Hohng S., Ha T. (2008). A practical guide to single-molecule FRET. Nat. Methods.

[21] Miyauchi Y.H., Chiashi S.H., Murakami Y., Hayashida Y., Maruyama S. (2004). Fluorescence spectroscopy of single-walled carbon nanotubes synthesized from alcohol. Chem. Phys. Lett..

[22] Chiu L.D., Su L., Reichelt S., Amos W.B. (2012). Use of a white light supercontinuum laser for confocal interference-reflection microscopy. J. Microsc..

[23] Selci S., Bertani F.R., Ferrari L. (2011). Supercontinuum ultra wide range confocal microscope for reflectance spectroscopy of living matter and material science surfaces. AIP Adv..

[24] Zhang J., Landry M.P., Barone P.W., Kim J., Lin S., Ulissi Z.W., Lin D., Mu B., Boghossian A.A., Hilmer A.J. (2013). Molecular recognition using corona complex made of artificial polymers adsorbed on carbon nanotubes. Nat. Nanotechnol..

[25] Hudson P.J. (1999). Recombinant antibody constructs in cancer therapy. Curr. Opin. Immunol..

[26] Trikha M., Yan L., Nakada M.T. (2002). Monoclonal antibodies as therapeutics in oncology. Curr. Opin. Biotechnol..

[27] Yang R., Jin J., Chen Y., Shao N., Kang H., Xiao Z., Tang Z., Wu Y., Zhu Z., Tan W. (2008). Carbon nanotube-quenched fluorescent oligonucleotides: Probes that fluoresce upon hybridization. J. Am. Chem. Soc..

[28] Sames D., Dunn M., Karpowicz R.J., Sulzer D. (2013). Visualizing Neurotransmitter Secretion at Individual Synapses. ACS Chem. Neurosci..

[29] Braak H., Ghebremedhin E., Rub U., Bratzke H., Del Tredici K. (2004). Stages in the development of Parkinson's disease-related pathology. Cell Tissue Res..

[30] Kim D., Koseoglu S., Manning B.M., Meyer A.F., Haynes C.L. (2011). Electroanalytical eavesdropping on single cell communication. Anal. Chem..

[31] Savtchenko L.P., Rusakov D.A. (2007). The optimal height of the synaptic cleft. Proc. Natl. Acad. Sci. USA.

[32] Kruss S., Landry M.P., Ende E.V., Lima B.M., Reuel N.F., Zhang J., Nelson J., Mu B., Hilmer A., Strano M. (2014). Neurotransmitter detection using corona phase molecular recognition on fluorescent single-walled carbon nanotube sensors. J. Am. Chem. Soc..

[33] Giraldo J.P., Landry M.P., Faltermeier S.M., McNicholas T.P., Iverson N.M., Boghossian A.A., Reuel N.F., Hilmer A.J., Sen F., Brew J.A. (2014). Plant nanobionics approach to augment photosynthesis and biochemical sensing. Nat. Mater..

[34] Iverson N.M., Barone P.W., Shandell M., Trudel L.J., Sen S., Sen F., Ivanov V., Atolia E., Farias E., McNicholas T.P. (2013). *In vivo* biosensing via tissue-localizable near-infrared-fluorescent single-walled carbon nanotubes. Nat. Nanotechnol..

[35] Bisker G., Iverson N.M., Ahn J., Strano M.S. (2014). A Pharmacokinetic Model of a Tissue Implantable Insulin Sensor. Adv. Healthc. Mater..

[36] Kelly K.L., Coronado E., Zhao L.L., Schatz G.C. (2002). The Optical Properties of Metal Nanoparticles: The Influence of Size, Shape, and Dielectric Environment. J. Phys. Chem. B.

[37] Shannahan J.H., Brown J.M., Chen R., Ke P.C., Lai X., Mitra S., Witzmann F.A. (2013). Comparison of nanotube-protein corona composition in cell culture media. Small.

[38] Zheng M., Jagota A., Strano M.S., Santos A.P., Barone P., Chou S.G., Diner B.A., Dresselhaus M.S., Mclean R.S., Onoa G.B. (2003). Structure-based carbon nanotube sorting by sequence-dependent DNA assembly. Science.

[39] Marega R., Aroulmoji V., Bergamin M., Feruglio L., Dinon F., Bianco A., Murano E., Prato M. (2010). Two-dimensional diffusion-ordered NMR spectroscopy as a tool for monitoring functionalized carbon nanotube purification and composition. ACS Nano.

[40] Dukovic G., Balaz M., Doak P., Berova N.D., Zheng M., McLean R.S., Brus L.E. (2006). Racemic single-walled carbon nanotubes exhibit circular dichroism when wrapped with DNA. J. Am. Chem. Soc..

[41] Choi J.H., Strano M.S. (2007). Solvatochromism in single-walled carbon nanotubes. Appl. Phys. Lett..

[42] Moore V.C., Strano M.S., Haroz E.H., Hauge R.H., Smalley R.E. (2003). Individually suspended single-walled carbon nanotubes in various surfactants. Nano Lett..

[43] Zhang J., Kruss S., Hilmer A.J., Shimizu S., Schmois Z., de la Cruz F., Barone P.W., Reuel N.F., Heller D.A., Strano M.S. (2013). A Rapid, Direct, Quantitative, and Label-Free Detector of Cardiac Biomarker Troponin T Using Near-Infrared Fluorescent Single-Walled Carbon Nanotube Sensors. Adv. Healthc. Mater..

[44] Ballauff M., Lu Y. (2007). “Smart” nanoparticles: Preparation, characterization and applications. Polymer.

[45] Granite M., Radulescu A., Cohen Y. (2012). Small-Angle Neutron Scattering from Aqueous Dispersions of Single-Walled Carbon Nanotubes with Pluronic F127 and Poly(vinylpyrrolidone). Langmuir.

[46] Danino D. (2012). Cryo-TEM of soft molecular assemblies. Curr. Opin. Colloid Interface Sci..

[47] Mielanczyk L., Matysiak N., Michalski M., Buldak R., Wojnicz R. (2014). Closer to the native state. Critical evaluation of cryo-techniques for Transmission Electron Microscopy: Preparation of biological samples. Folia Histochem. Cytobiol..

[48] Berlepsch H.V., Ludwig K., Schade B., Haag R., Böttcher C. (2014). Progress in the direct structural characterization of fibrous amphiphilic supramolecular assemblies in solution by transmission electron microscopic techniques. Adv. Colloid Interface Sci..

[49] Wang H., Zhou W., Ho D.L., Winey K.I., Fischer J.E., Glinka C.J., Hobbie E.K. (2004). Dispersing Single-Walled Carbon Nanotubes with Surfactants: A Small Angle Neutron Scattering Study. Nano Lett..

[50] Sánchez-Castillo A., Román-Velázquez C.E., Noguez C. (2006). Optical circular dichroism of single-wall carbon nanotubes. Phys. Rev. B.

[51] Samsonidze G.G., Grüneis A., Saito R., Jorio A., Filho A.G.S., Dresselhaus G., Dresselhaus M.S. (2004). Interband optical transitions in left- and right-handed single-wall carbon nanotubes. Phys. Rev. B.

[52] Panhuis M., Sainz R., Innis P.C., Kane-Maguire L.A.P., Benito A.M., Martínez M.T., Moulton S.E., Wallace G.G., Maser W.K. (2005). Optically Active Polymer Carbon Nanotube Composite. J. Phys. Chem. B.

[53] Yokoyama A., Yoshida M., Ishii A., Kato Y.K. (2014). Giant Circular Dichroism in Individual Carbon Nanotubes Induced by Extrinsic Chirality. Phys. Rev. X.

